# Automatic Measurement of Frontomaxillary Facial Angle in Fetal Ultrasound Images Using Deep Learning

**DOI:** 10.3390/s25030633

**Published:** 2025-01-22

**Authors:** Zhonghua Liu, Jin Wang, Guorong Lyu, Haisheng Song, Weifeng Yu, Peizhong Liu, Yuling Fan, Yaocheng Wan

**Affiliations:** 1 Department of Ultrasound, Quanzhou First Hospital Affiliated to Fujian Medical University, Quanzhou 350122, China; yuweifengab@163.com; 2 School of Physics and Electronic Engineering, Northwest Normal University, Lanzhou 730070, China; jinwang202406@163.com (J.W.); songhs@nwnu.edu.cn (H.S.); 3 Department of Ultrasound, Second Affiliated Hospital of Fujian Medical University, Quanzhou 362000, China; 4 School of Engineering, Huaqiao University, Quanzhou 362021, China; pzliu@hqu.edu.cn (P.L.); yling_fan@126.com (Y.F.); 5 School of Electronic and Information Engineering, Changchun University of Science and Technology, Changchun 130022, China; wyc202412@163.com

**Keywords:** automatic measurement, deep learning, semantic segmentation, ultrasound image

## Abstract

Accurate measurement of frontomaxillary facial (FMF) angles in prenatal ultrasound (US) scans plays a pivotal role in the screening of trisomy 21. Nevertheless, this intricate procedure heavily relies on the proficiency of the ultrasonographer and tends to be a time-intensive task. Furthermore, FMF angles are subjective when measured manually. To address this challenge, we propose a deep learning-based assisted examination framework for automatically measuring FMF angles on 2D ultrasound images. Firstly, we trained a deep learning network using 1549 fetal ultrasound images to achieve automatic and accurate segmentation of critical areas. Subsequently, a key point detection network was employed to predict the coordinates of the requisite points for calculating FMF angles. Finally, FMF angles were obtained through computational means. We employed Pearson correlation coefficients and Bland–Altman plots to assess the correlation and consistency between the model’s predictions and manual measurements. Notably, our method exhibited a mean absolute error of 2.354°, outperforming the typical standards of the junior expert. This indicates the high degree of accuracy and reliability achieved by our approach.

## 1. Introduction

Trisomy 21, commonly referred to as Down syndrome (DS), is a prevalent chromosomal abnormality that often manifests in cognitive impairment, facial dysmorphism, delayed growth, developmental delays, and a diverse array of congenital malformations. While people without DS have 46 chromosomes, people with DS have one more chromosome 21, and the extra chromosome monosomy affects their physical and intellectual development [1]. In a study, it was established that there is a correlation between the occurrence of DS and the age of pregnancy. Specifically, the probability of this condition increases concomitantly with advancing maternal age [1]. DS is an incurable lifelong condition that significantly affects individuals’ health, so early detection helps families and wider society to prepare for this. Blood tests and ultrasound can be performed in early- or mid-pregnancy for DS. Blood testing is not a conclusive diagnostic tool for DS, but it can flag a heightened risk of the condition.

Meanwhile, more invasive procedures like amniocentesis and chorionic villus sampling (CVS) offer a more definitive diagnosis, albeit with a slight risk of complications that may potentially result in miscarriage. Ultrasonography is a non-invasive method, as patients with DS often have specific facial features that ultrasound can observe [2,3]. With the continuous development of ultrasound medical technology, ultrasound-based fetal facial examination is the most intuitive and effective means of clinical examination. Ultrasound is a non-invasive, non-radioactive, painless, and affordable technology that can visualize facial disorders by receiving echo information from the fetus’ face and its collateral structures. Ultrasound is a safe, radiation-free test that causes no harm even when performed multiple times during pregnancy [3,4]. It has been the first choice for prenatal screening of fetuses for decades.

**Figure 1:**
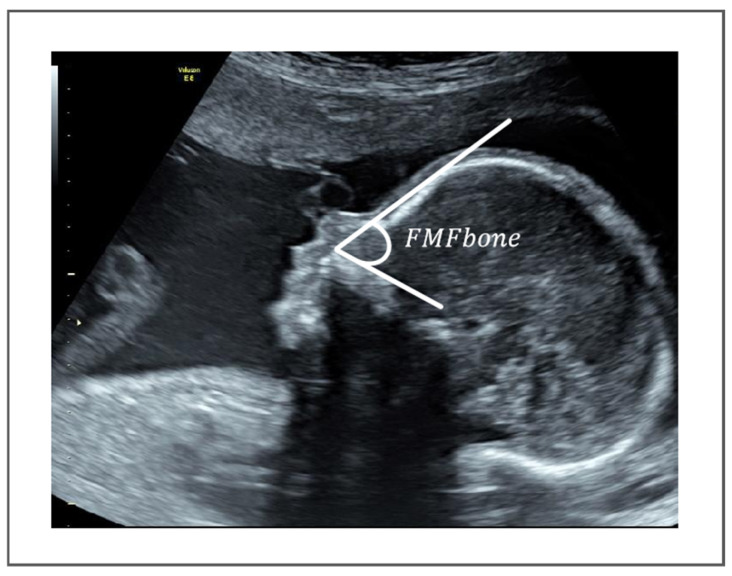
Measurement method of the FMFbone.

**Figure 2:**
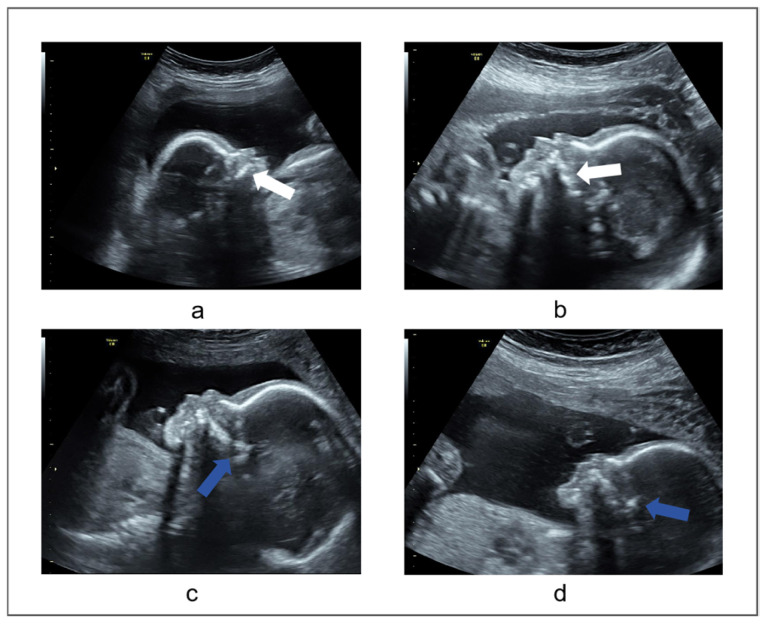
(**a**) Ambiguous maxillary structure. the white arrows indicate the definition of the palatal margin might become indistinct. (**b**) Irregular maxillary structure, Indicated by the white arrow (**c**) Thickening of the posterior part of the maxilla. The blue arrow points it out. (**d**) Irregularity of the posterior part of the maxilla, As shown by the blue arrow.

Recent research has emphasized the potential utility of measuring the FMF angle as a supplementary screening tool for trisomy 21 [5]. Moreover, ultrasound evaluations during pregnancy have revealed that fetuses with DS exhibit shorter maxillary lengths and shallower depth in comparison to haploid fetuses [6]. In fetuses with trisomy 21, the FMF angle is notably elevated compared to that observed in haploid fetuses [7]. The study’s primary metric, termed FMFbone, represents the angle formed between the superior margin of the upper palate and the bony forehead. As depicted in Figure 1, the first ray traverses the anterior section of the upper palate, commencing from the superior margin of the maxilla, while the second ray originates from the apex along the frontal bone, defining the FMFbone angle. The measurement of FMF angle remains a challenging task with three main difficulties, as shown in Figure 2:

- Poor image quality may affect the doctor’s judgment. Due to the complex interaction between US waves and the biological tissues of the mother and fetus, ultrasound images can be affected by speckle noise, acoustic shadow, motion blurring, missing boundaries, and poor picture clarity. This situation can affect the physician’s measure result. For instance, the definition of the palatal margin might become indistinct, evident from the white arrow depicted in Figure 2a,b;
- Judgment of critical structures on ultrasound images is more difficult. The anterior segment of the palate stands out as the most prominent, while the posterior region might exhibit thickening and irregularity, as illustrated by the blue arrow in Figure 2c,d, which can affect the physician’s judgment;
- Manual measurement inherently incorporates a degree of subjectivity. The subjective nature of manual measurement can be problematic. The poor performance of manual measurement can be attributed to the lack of standardized training.

Prior investigations have demonstrated the remarkable capabilities of deep learning in attaining cutting-edge results across the domains of computer vision and biomedical measurement [8]. Deep learning has proven to be an effective tool for processing medical images. Ref. [9] completed the measurement of the FMF angle during the first trimester. Chen et al. [10] successfully implemented an automated approach for measuring the width of the fetal lateral ventricle (LV) in 2D ultrasound images. The methodology achieved an average absolute measurement error of 1.8 mm, outperforming manual measurement techniques. Van et al. [11] proposed a multitask learning network, MRNet, which implements segmentation of the lumbar spine and automatic parameter prediction tasks for lumbosacral spine examination. Hyun et al. [12] used deep learning for the first time to provide an automated assessment of amniotic fluid index on ultrasound images to provide an automated measurement of amniotic fluid index (AFI). Jang et al. [13] used a convolutional neural network to classify the image and then used the Hough transform method to automatically measure the abdominal circumference (AC); following acceptance check experiments, the accuracy level obtained with the expert was 0.809. Pluym et al. Ref. [14] aims to compare the accuracy of measurements of fetal intracranial structures using the artificial intelligence tool SonoCNS Fetal Brain with manual measurements. This device comes from the General Electric Company (Chicago, IL, USA). The research results indicate that automated measurements exhibit good reliability (with Intraclass Correlation Coefficients, ICCs, ranging from 0.80 to 0.88) compared to manual measurements for biparietal diameter (BPD) and head circumference (HC). Li et al., in [15], introduced a variety of novel biometric markers, including biparietal diameter (BPD) and occipitofrontal diameter (OFD). Deep learning is widely used in ultrasound images [16,17,18,19,20]. Computer-aided diagnostic techniques remove the need for manual tracing and could both increase efficiency and improve reproducibility. FMF angles are usually measured manually by ultrasonographers. This requires a high level of medical expertise and clinical experience. Subjectivity makes it difficult to perform the examination in primary hospitals. To efficiently harness the time and expertise of specialized physicians, we assert that cutting-edge artificial intelligence technology is imperative for automating biometric measurements. This automation not only enhances speed and reproducibility, but also elevates measurement accuracy. Implementing automated measurement techniques holds paramount importance in refining the prenatal diagnosis workflow and enhancing its precision.

In this paper, we propose a novel deep learning-driven measurement method for the first time, with the aim of designing an automated approach for measuring the FMF angle using sagittal ultrasound images of 1549 fetuses during the second trimester for examination and diagnosis. The proposed methodology is founded upon an advanced segmentation network, which is capable of efficiently segmenting the ultrasound images. This segmentation process is then utilized to predict the coordinates of the points necessary for angle measurement, and subsequently, to calculate the angle. During the experimental phase, physicians manually annotated the training data at the pixel level, which were then used to train our deep learning network. The experimental results demonstrate that the proposed method achieves a high level of accuracy. The primary contributions of this study are as follows:

- This paper presents an innovative approach to FMF angle measurement in fetuses during the second trimester, employing a novel automated method based on deep learning.
- This method accelerates the process of prenatal diagnosis and enhances the accuracy of the diagnosis.
- The accuracy of our method is validated through statistical analysis of consistency with senior experts.

## 2. Materials and Methods

**Figure 3:**
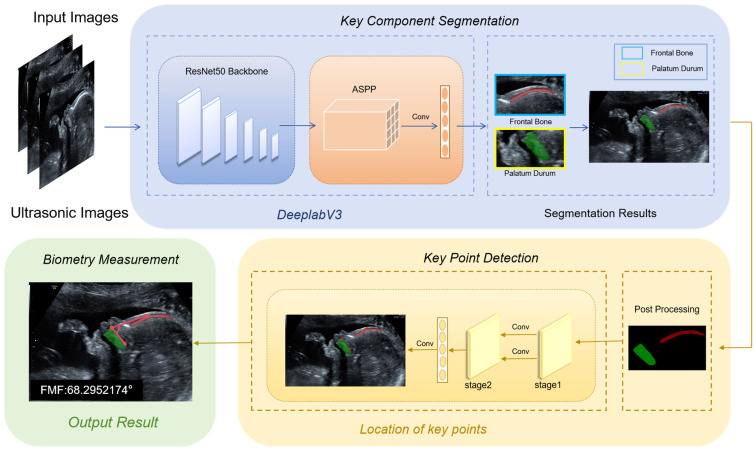
This study proposes a block diagram of the process of automatic measurement of the FMF angle, which demonstrates the measurement process through AI models Firstly, the segmentation is performed by Key Component Segmentation, then the Key Point Dection module is used for key point detection, and finally the angle is calculated using Biometry Measurement.

In this study, we proposed a method for automatic FMF angle measurement, divided into three steps: segmentation, prediction, and measurement. The method proposed in this study is illustrated in Figure 3. Initially, the segmentation process commences with the utilization of the key component segmentation module, specifically targeting the fetus’s palate and frontal bone regions. Then, on the obtained segmentation map, the key point detection module is used to predict the three key points needed to measure the fetal frontal maxillary angle. Finally, the angle is automatically measured using the biometry measurement module.

### 2.1. Study Population

This retrospective study utilized archived fetal images of singleton pregnancies obtained by mid-trimester ultrasound scanning between February 2020 and October 2022 at the Quanzhou First Hospital, affiliated to Fujian Medical University. For analysis, two-dimensional images depicting the median sagittal plane of the fetal face were chosen, spanning the gestational period between 14 and 27 weeks.

The inclusion criteria were as follows:

- We selected a median sagittal image of the fetal face, encompassing both the entire head and upper thorax, where these two regions account for over 65% of the total image area;
- The fetal face display is complete and clear. The ultrasonic images include the forehead, nasal bone, nasion, palate, mandible, chin, and upper/lower lip;
- The fetal image is not obscured by the umbilical cord or limb.

Individuals with blurred images, rendering fetal facial structures indistinct, were excluded from the study. The data employed in this research were rendered anonymous, ensuring adherence to ethical standards and alleviating any potential concerns.

### 2.2. Data Annotation

The COCO-Annotator tool can be employed to annotate key points for the key point identification task of the key point detection module. COCO-Annotator, a web-oriented tool tailored specifically for computer vision researchers and practitioners, serves as an efficient means for annotation. Utilizing the Labelme (Version 4.5.9) tool, the senior expert manually annotated key anatomical structures on 1549 fetal ultrasound images at pixel-level precision. This annotation process served as the foundation for executing the semantic segmentation task within the critical component segmentation module. Labelme, an image annotation tool originating from the Computer Science and Artificial Intelligence Laboratory (CSAIL) at the Massachusetts Institute of Technology (MIT), is crafted in Python and PyQT. It enables the precise annotation of images in various forms, including polygons, rectangles, circles, polylines, line segments, and dots. Labelme’s versatility is evident in its numerous image segmentation tasks.

### 2.3. Deep Learning Model Architecture

This research aims to devise an automated biometric system, employing a deep learning technique for accurately gauging FMF angles through the utilization of fetal ultrasound imagery. The method proposed in this study is designed to measure the FMF angle. The automated measurement of FMF angle frames is structured into three distinct subtasks, which are visualized in Figure 3. As an initial step, the ultrasound image undergoes semantic segmentation. Subsequently, the segmentation network model undergoes a learning process to extract pertinent features from the image, ultimately identifying two crucial anatomical structures: the frontal bone and the palate. Then, the acquired anatomical structure images are trained a second time using a keypoint detection network to obtain the points needed to measure the angle of FMF, which we proposed according to HRnet [21] to obtain the points required to measure the FMF angle. Finally, the FMF angle is calculated based on the pixel points.

The key component segmentation module employs the DeepLabV3 [22] segmentation network to delineate the key structures of the fetal face, resulting in the classification of three categories: palate, frontal bone, and background. The utilization of deep convolutional neural networks (DCNNs) in semantic segmentation tasks often reduces spatial resolution. Ref. [23] shows that in semantic segmentation, tasks result in a decrease in spatial resolution. This is because deep convolutional neural networks undergo consecutive pooling or convolution operations. While these operations increase the receptive field of the DCNNs, they also generate the phenomenon of information loss during the downsampling processes.

**Figure 4:**
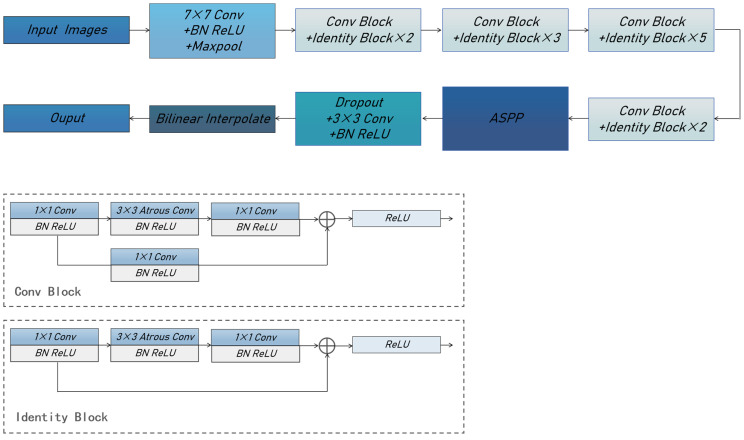
An end-to-end segmentation model is adopted in this study. The architecture comprises our network model with three modules: the identity block, the transformation block, and the atrous spatial pyramid pooling (ASPP) block.

**Figure 5:**
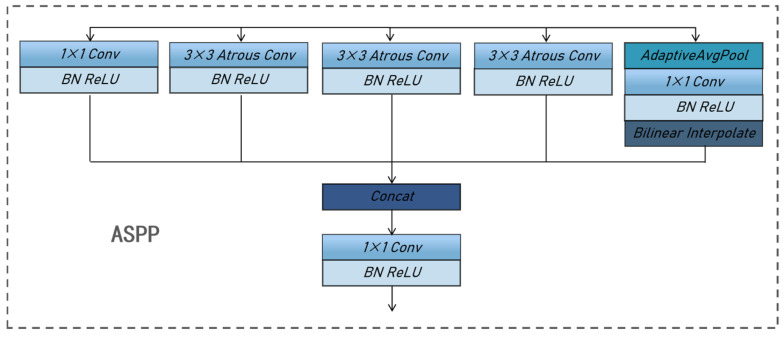
ASPP structure diagram.

To mitigate the challenge of information loss stemming from downsampling, DeepLabV3 introduces atrous convolution, a technique that enlarges the receptive field and facilitates the acquisition of multi-scale contextual data. This approach has been demonstrated to be effective for semantic image segmentation [24]. Atrous convolution, also known as dilation convolution or inflationary convolution, expands the receptive field by adding voids and introduces a new hyperparameter, the dilation rate, to the convolutional layer. The dilation rate dictates the spacing of values during the convolution kernel’s data processing. By adjusting this hyperparameter, atrous convolution effectively expands the receptive field to varying degrees, ultimately enhancing the precision of segmentation outcomes. This approach enables capturing multi-scale contextual information, thereby optimizing segmentation accuracy. Figure 4 illustrates the structure of our segmentation network, which comprises an input layer with dimensions of 480 × 480 × 3. The features are extracted using ResNet [25] as the backbone, after which the image is downsampled by a factor of 8. This is followed by an atrous spatial pyramid pooling (ASPP) structure, which applies atrous convolution with different rates on the feature map. The ASPP algorithm employs a different rate of atrous convolution and is an efficient method for capturing multi-scale information. Its structure is shown in Figure 5. This network architecture consists of five parallel branches, encompassing a 1 × 1 convolutional layer, three 3 × 3 atrous convolutional layers, and a global average pooling layer. Notably, the dilation rates for the atrous convolutions are set to 12, 24, and 36, respectively, to achieve optimal performance. The global average pooling layer increases the global context information, followed by a 1 × 1 convolutional layer. Finally, a bilinear interpolation method is employed to incorporate global context information, after which the outputs of the five branches are obtained by converting the five. Subsequently, the outputs from the five branches are concatenated, and a convolutional layer is utilized to integrate the information from each individual branch. This is followed by a reduction to the original size by bilinear interpolation. The cross-entropy loss function is employed to address the loss incurred by the segmentation network. By referencing the weight parameter, we can reduce the weight assigned to the background, thereby improving the issue of class imbalance. The specific formula is shown in (1).

(1) $$Loss=-\sum_{i=1}^{n}\omega \times P(i)\times \log_{2}Q(i)$$

where *i* denotes the index of the class, *P*(*i*) represents the value of the *i*-th class in the true label, *Q*(*i*) represents the probability predicted by the model for the *i*-th class, *ω* is the weight, and *n* is the number of categories.

In the key point detection module, we predict two crucial points: the upper edge of the palate and the outermost point of maximum anterior deviation on the frontal bone’s exterior surface. Algorithms for keypoint detection can be classified according to the output format of the model into regression and heatmap methods. Utilizing the regression approach, the input image is introduced into a convolutional network. Upon processing through a fully connected layer, this network subsequently generates the coordinates of the key points. This method has proven effective in [26,27]. The heatmap-based supervised model [28,29] learns a Gaussian probability distribution map, which renders each point in the ground truth as a Gaussian heatmap. Finally, the network outputs a heatmap of size $W^{\prime }\times H^{\prime }$, $\{H_{1},H_{2}$,…,$H_{k}\}$, where $H_{k}$ is the location confidence of the kth keypoint. k feature maps correspond to N keypoints. The HRnet method is a technique for predicting keypoints based on heat maps, it is one of the most popular methods in existing research, parallel connection resolution from high to low subnetworks can maintain high resolution throughout the training process. It entails the generation of a heat map for each keypoint, with the subsequent selection of the location with the highest heat values as the keypoint. HRNet comprises four modules, with each module comprising a connection part and a core idea of continuously fusing information on different scales.

**Figure 6:**
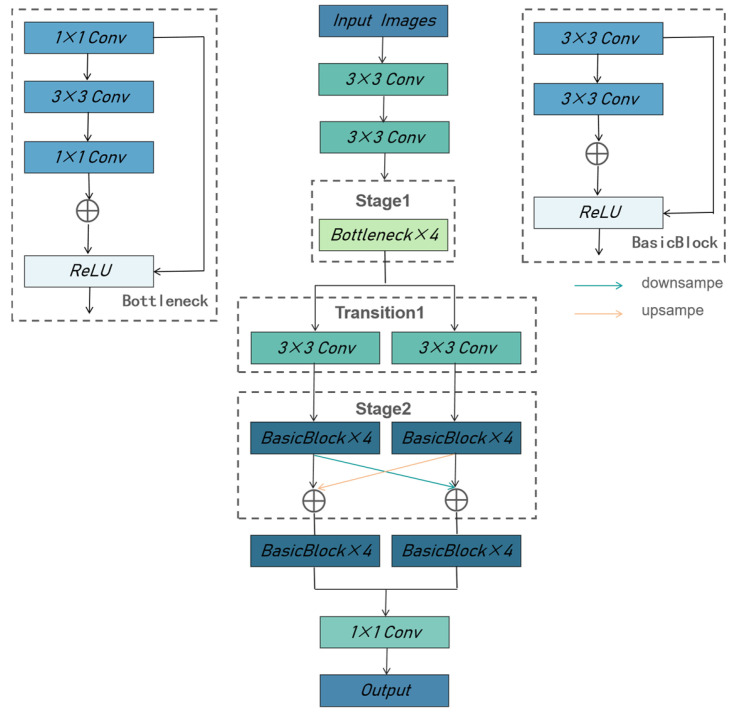
Structure of the keypoint detection network. The green arrow represents downsampling, and the orange arrow represents upsampling.

As illustrated in Figure 6, we built two parallel sub-network branches based on the idea of fusing different scales. The network was initially downsampled four times through two convolutional layers of size 3 × 3 with a step size of two, then with four bottlenecks. For each scale branch, the stage structure fuses the information on different scales by downsampling and upsampling. To achieve downsampling, a convolutional layer with a 3 × 3 kernel and stride of 2 is employed. Upsampling is accomplished through nearest neighbor interpolation. Subsequently, a 1 × 1 convolutional layer with an applied convolutional kernel produces the feature layer, or heatmap, corresponding to each key point. The network undergoes training utilizing mean squared error loss, as expressed in (2).

(2) $$Loss=\frac{1}{n}\sum_{i=1}^{n}{\left(P(i)-Q(i)\right)}^{2}$$

Q(i) and P(i) represent the model’s predicted value and true sample value, respectively, and *n* is the number of categories.

**Figure 7:**
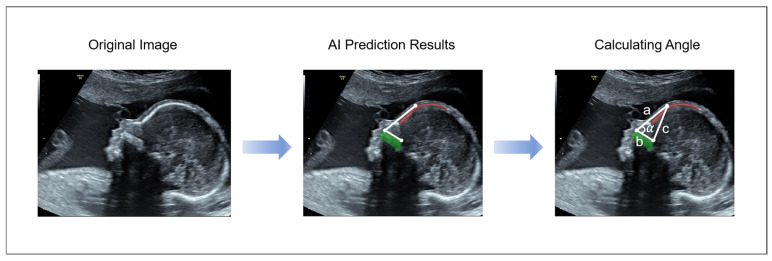
General flowchart of the biometry measurement module. The angle is calculated from the coordinates of the points.

The third step is the biometry measurement module. This module first obtains the coordinates of the keypoints predicted by the keypoint detection network and forms a triangle through the three keypoints. It then calculates the lengths of the three sides of the triangle using (3), where ${\;x}_{1}$, $x_{2}$, $y_{1}$, and $y_{2}$ are the horizontal and vertical coordinates of the endpoints of the corresponding line segments, respectively. *H* is the length of the side of the triangle formed. As depicted in Figure 7, the radian value corresponding to the angle associated with each side of the triangle is subsequently determined using the inverse cosine function. Specifically, the lengths of the three sides of the triangle are represented by the values *a*, *b*, and *c*, while *θ* signifies the radian value of the desired angle. The specific formula is shown in (4). Finally, the radian values are converted to angle values using the degrees function in the math library. The specific flow is shown in Figure 7.

(3) $$H=\sqrt{{\left(x_{1}-x_{2}\right)}^{2}+{\left(y_{1}-y_{2}\right)}^{2}}$$

(4) $$\theta =a\cos\frac{b^{2}+c^{2}-a^{2}}{2bc}$$

## 3. Experiments

In accordance with the established criteria for inclusion and exclusion, a total of 1549 mid-trimester fetal ultrasound images were obtained. From this dataset, 116 fetal cases were randomly selected to constitute the validation set, while the remaining dataset was divided into a training set and a test set in a 9:1 ratio. The training set was utilized for the construction and training of the model, and the test set provided an independent evaluation of its performance. The validation set was used to adjust the model hyperparameters and monitor for overfitting. The model was trained on a computer with a 3.8 GHz Intel i7-10700K CPU and an NVIDIA GeForce RTX 2060 GPU, which produced by NVIDIA Corporation (Santa Clara, CA, USA). The model was constructed using the PyTorch (Version 1.10) deep learning framework on the Windows 10 operating system. We empirically determined the hyperparameter settings based on our experiences. The initial learning rate for the semantic segmentation model was set to 2 × 10^−3^ with a batch size of four and a polynomial learning rate strategy. The input images were resized to 480 × 480, and the semantic segmentation network was pre-trained using the COCO dataset. During the training phase, data augmentation was employed to randomly scale the images between 0.5 and 2, and flipping and random cropping were performed to ensure the generalization performance of the network. The mean and standard deviation values used for pre-training normalization were [0.163, 0.178, 0.193] and [0.185, 0.199, 0.208], respectively. The SGD optimizer was used with a momentum parameter set to 0.9 and a weight decay of 1 × 10^−4^. The network was trained using cross-entropy loss over 200 epochs. For the keypoint detection network, the initial learning rate was set to 3 × 10^−3^, with a batch size of 16. Data augmentation techniques, including scaling, rotation, and resizing to a fixed size, were applied to the dataset. The scaling range was between 0.65 and 1.35, and the rotation angle was between −45 degrees and 45 degrees. Image tensors were normalized using specified mean values [1.295, 1.467, 1.659] and standard deviations [0.305, 0.401, 0.466]. The AdamW optimizer was utilized with β1 set to 0.9, β2 set to 0.999, ∈ set to 1 × 10^−8^ and a weight decay of 1 × 10^−4^. The network was trained using mean squared error loss over 100 epochs.

### 3.1. Evaluation of Segmentation

To assess the efficacy of the semantic segmentation model, three pivotal metrics were chosen: pixel accuracy (PA) [30], intersection over union (IOU) [31], and dice coefficient (Dice) [32]. PA captures the proportion of accurately classified pixels among all pixels. IOU measures the overlap between predicted and ground truth pixel values. Meanwhile, Dice serves as a measure of how closely the model’s predicted segmentation results align with the actual labels.

(5) $$PA=\frac{TP+TN}{TP+TN+FP+FN}$$

(6) $$IOU=\frac{TP}{TP+TN+FN}$$

(7) $$Dice=\frac{2TP}{2TP+FP+FN}$$

**Figure 8:**
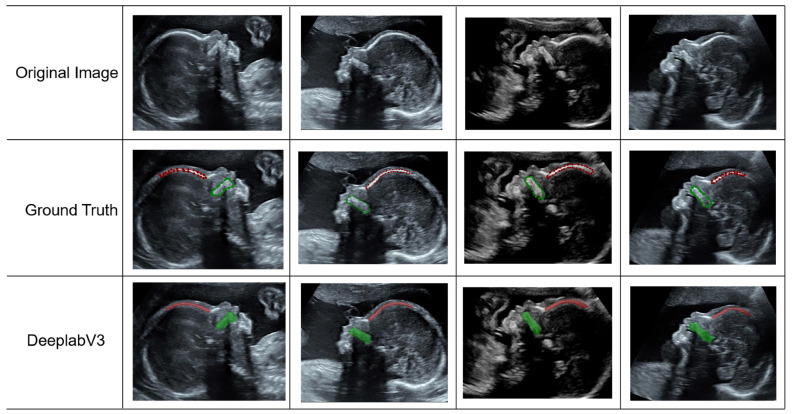
Fetal facial ultrasound images, from top to bottom, the first row is the original image, the second row is the annotated ground truth, and the third row is the segmentation result of DeepLabV3.

In (5)–(7) *TP* signifies the count of instances where the model correctly forecasts a positive outcome as truly positive; *TN* represents the count of cases where the model accurately predicts a negative instance as negative; *FP* denotes the number of occurrences where the model wrongly classifies a negative sample as positive; and *FN* refers to the number of instances where the model incorrectly predicts a positive sample as negative. Table 1 presents the results of our experiments in segmenting specific anatomical structures using DeepLabV3. The model performance was evaluated using IOU, PA, and Dice. Utilizing the DeepLabV3-based segmentation model, our dataset achieved remarkable accuracy. Specifically, for the palate and frontal bone, the precision–recall (PA) scores were 91.6 and 95.1, respectively. Furthermore, the intersection over union (IOU) values were 62.9 and 70.3, while the Dice similarity coefficients were 77.3 and 82.5 for the palate and frontal bone. Table 2 shows the average Dice values for the fivefold cross-validation experiments. The automatic segmentation based on DeepLabV3 achieves high accuracy on cross-validation of our dataset, with average Dice of 77.3 ± 1.830 and 82.4 ± 0.923 for the palate and frontal bone, respectively. Figure 8 presents the segmentation outcomes of the DeepLabV3 model, juxtaposed with the ground truth data, for a comparative analysis.

### 3.2. Automatic Biometric Measurements Evaluation

The model is validated using external data. Firstly, the data were tested for normal distribution. If the measurements adhered to a normal distribution, we employed the mean absolute error (MAE), mean relative error (MRE), and standard deviation (SD) as metrics to assess their accuracy. Conversely, in cases where the measurements did not conform to a normal distribution, we represented them using the median and range. The MAE is expressed as the mean of the absolute value of the error. The MRE, standing for mean relative error, represents the ratio of the error to the true value. (8) and (9) illustrate the specific methodology for its calculation. Manual measurements conducted by the senior expert were employed as a reference standard. To evaluate the correlation between manual and automated measurements, we employed Pearson’s correlation coefficient as well as the interclass correlation coefficient (ICC) with the “two-way stochastic” and “absolute agreement” variants. Furthermore, we utilized Bland–Altman plots to analyze the agreement between the two methodologies.

**Figure 9:**
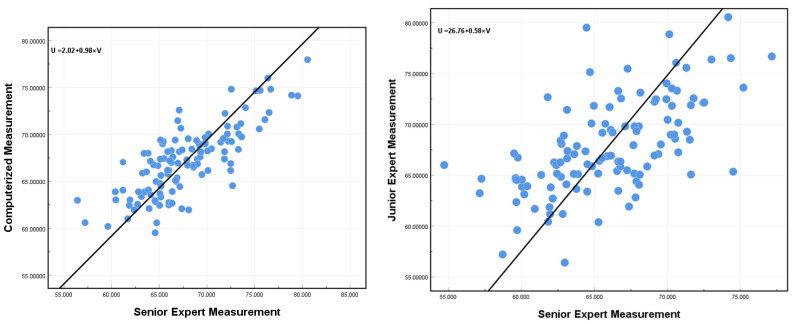
Pearson correlation coefficient plots showing the agreement of the measurements between the senior expert and AI are shown on the left, and the junior expert and the senior expert on the right. In the figure, a circle represents a single fetus, and the straight line is the fitting line of the points.

**Figure 10:**
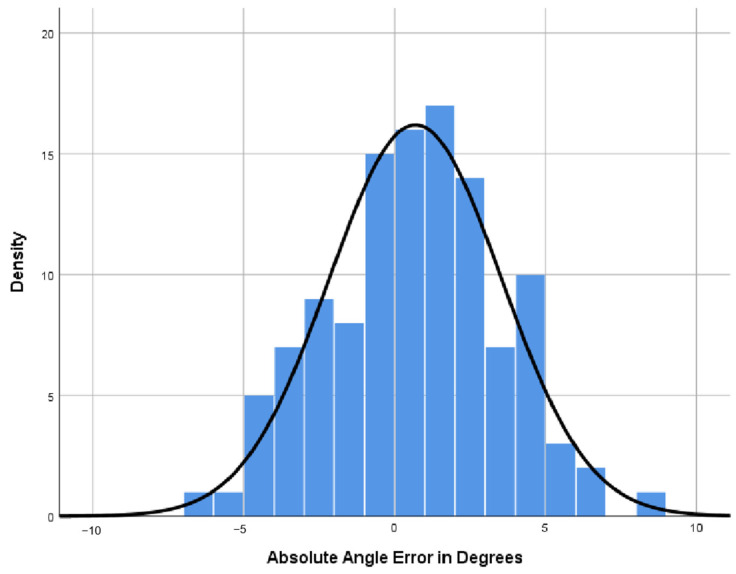
Histogram of the error distribution, The horizontal axis is the difference between the senior expert and AI, and the vertical axis is the frequency of the difference. The plot reveals that the error distribution is approximately centered around zero, with no apparent skewness in the errors.

A comparison was conducted between the results of the senior expert and the proposed automated measurement method, with the results presented in Table 3. The AI group demonstrated satisfactory performance on the FMF angles measures. The mean absolute error (MAE) and mean relative error (MRE) both exhibited low values, specifically 2.354 and 0.35, respectively, accompanied by a minimal variance. Furthermore, the mean values were very close to the senior expert’s. The AI measurements of FMF angles in the clinical validation set were 67.352° ± 3.877, while the senior expert’s measurements were 67.790° ± 4.609. Finally, the mean measurement deviations were 0.686° ± 2.85, and the significance level was *p* < 0.001. The comparative analysis of measurements between the junior expert and the senior expert is summarized in Table 4. In the validation set, the measurements of the junior expert were 65.922 ± 4.363, with mean measurement deviations of −1.869 ± 3.967 and MAE and MRE values of 3.428 and 0.050, respectively. The MAE and MRE metrics are higher than those observed between AI and the senior expert, indicating that the proposed method is more accurate than that employed by the junior expert, as illustrated in Figure 9. The distribution of errors is displayed in Figure 10.

(8) $$MAE=\frac{1}{n}\sum_{i=1}^{n}\left|{y_{i}}^{\prime }-y_{i}\right|$$

(9) $$MRE=\frac{1}{n}\sum_{i=1}^{n}\left|\frac{{y_{i}}^{\prime }-y_{i}}{y_{i}}\right|$$

To further evaluate the extent of correlation and concordance between the senior expert and AI, we undertook a comparative analysis of the FMF angle measurements’ ICC reliability indices. Table 5 presents the detailed ICC data. Specifically, the ICC value comparing AI’s automated measurements to the senior expert’s was 0.760, whereas the ICC comparing junior expert’s measurements to the senior expert’s stood at 0.562. It can be observed that the junior expert had significantly lower ICC values for the FMF angle measurements than the AI group. It is postulated that the reason for this phenomenon is that speckle noise, boundary-blurring, and complex anatomical structures in ultrasound images influence the judgment of junior physicians. In conclusion, the aforementioned analyses indicate that AI can not only relieve the experienced senior expert of the arduous task of manual measurements, but can also facilitate the advancement of junior experts’ diagnostic accuracy.

### 3.3. Bland–Altman Agreement Evaluation

**Figure 11:**
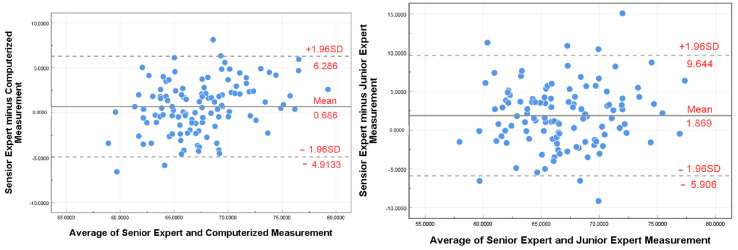
Bland–Altman analysis plot with measurements of the AI versus the senior expert on the left and the junior expert versus senior expert analyzed on the right. The solid line in the figure is the mean value of the difference, and the dotted line is 95% of the confidence interval.

To enhance the visualization of the concordance between the AI, senior expert, and junior expert measurements, we created Bland–Altman plots. These plots feature a solid central line that represents the average deviation between the two measurement sets, and upper and lower dashed lines that mark the 95% agreement intervals, respectively. This visualization approach aids in understanding the level of agreement between the various measurement methods. Figure 11 presents the results of the Bland–Altman plot analysis of the validation set. The AI measurements exhibited a substantial level of concurrence with the senior expert’s reading, as evidenced by a difference of 0.686, which falls within a 95% confidence interval, ranging from −4.9133 to 6.286. Additionally, the difference between the junior expert and senior expert measurements was 1.869 (95% confidence interval: −5.906 to 9.644).

## 4. Discussion

**Figure 12:**
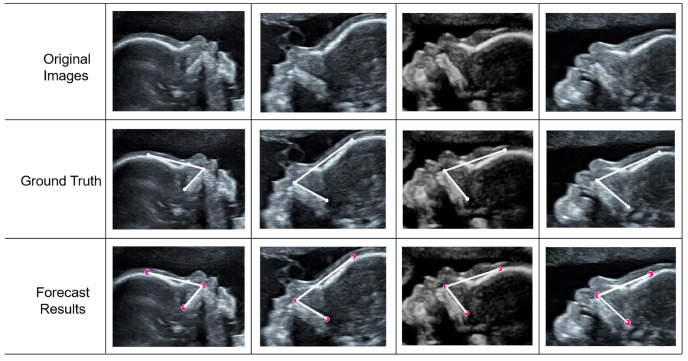
FMF measurement results. From top to bottom, the first row shows the original image, the second row shows the annotated ground truth, and the third row shows the results of the FMF prediction.

This study proposes an AI model that employs a deep learning approach to complete the FMF angle measurement task in mid-pregnancy. This is achieved using three modules: key component segmentation, key point detection, and biometry. The modules were designed to achieve the accurate segmentation of key structures, the accurate prediction of key points, and automatic measurement tasks, respectively. The advanced deep learning method was employed to achieve accurate segmentation of two structures, the palate and the frontal bone. The accuracy of the measurement is heavily influenced by the outcome of the segmentation process. Based on the accurate segmentation, three key points were accurately predicted using a keypoint detection algorithm. The findings from our experiments revealed a significant correlation between the proposed model and the assessments provided by senior expert, as illustrated in Figure 9. The Bland–Altman analysis (Figure 11) revealed a high degree of agreement between the computer-predicted and manually measured values for the FMF angle measurements. Figure 12 illustrates the comparison between AI and the senior expert. The results of this study may enhance the efficiency of sonographers and accelerate the process of clinical assessment of the fetus.

In late 2011, the United States introduced noninvasive prenatal testing utilizing massively parallel sequencing of cell-free DNA (cfDNA testing) in maternal plasma into its clinical prenatal care practices. The positive predictive values (PPV) for trisomy 21 were 45.5% (95% CI, 16.7 to 76.6) with cfDNA testing, as reported in [33]. Although it has been demonstrated that trisomy 21 is associated with certain sonographic markers, including increased nuchal fold thickness, a shortened femur, hyperechogenic bowel, or hydronephrosis, these distinctive characteristics have been noted in a limited number of fetuses, as reported in [34]. In contrast, using ultrasound to evaluate the FMF angle represents a highly sensitive approach [6]. The automation of biometry using AI has been demonstrated in numerous studies [35]. Automated biometric systems have the capability to reduce the impact of both intra- and inter-operator variability, thus improving the precision of diagnostic assessments. In this study, an automated FMF angle measurement model was constructed for the first time, which reduces the workload of ultrasonographers and simultaneously facilitates the development of an intelligent healthcare system.

This study is the first to propose the use of deep learning for automatic FMF angle measurement, which includes 1549 normal fetal images for model development and validation. In clinical applications, traditional manual measurement methods necessitate the involvement of a trained sonographer. This process is often challenging and time-consuming. The method improves the interpretability of the diagnostic process by automatically segmenting key structures and locating landmarks. It should be noted that this study has certain limitations. First of all, the ultrasound images used for training and validation were obtained from normal fetuses, and the applicability of the method to abnormal fetuses has not been tested. Secondly, this study is retrospective in nature. To ensure the veracity of AI models, it is advisable to conduct further prospective investigations in the future. Another significant challenge pertains to device variability, as ultrasound machines from different manufacturers may produce images with varying resolutions and qualities. This has the potential to impact the efficacy of the deep learning models, which were trained on a particular dataset of ultrasound images. To address this challenge, future research should involve testing the framework on images acquired from a diverse range of ultrasound devices to ensure its robustness and generalizability. Furthermore, consideration must be given to the real-time capability of the measurement methodology. The incorporation of deep learning models into ultrasound machines is a potential solution to this issue, as it would enable the real-time processing and analysis of images. In the domain of deep learning, the transferability of a model across different datasets is a pivotal consideration, as it determines the model’s performance on unseen data. The data presented herein were obtained from two distinct ultrasound instruments, namely the Philips EPlQ5 ultrasound machine and the GE Voluson E8 ultrasound machine. These two pieces of equipment originate from Philips (Amsterdam, The Netherlands) and General Electric Company (Boston, MA, USA), respectively. The dataset under consideration is diverse, and the model was trained using a training set comprising multiple data types. This approach facilitates the model’s learning of richer feature representations, thereby enhancing its transferability. It has been observed that certain multi-adversarial learning methods have shown considerable potential in enhancing the generalization ability of the model. Ref. [36] proposed a Multi-Adversarial Open-Set Domain Adaptation Network (MAOSDAN), which effectively addressed the problem of partial overlap between the label space of the source domain and the target domain. Although these methods have not yet been included in our research, they provide new concepts for future research work. In subsequent studies, we aim to integrate these techniques into our model to enhance its performance and generalization capability.

## 5. Conclusions

This study proposes a deep learning-based artificial intelligence-assisted measurement method for the fast and accurate automatic measurement of FMF angles. This method comprises three steps: segmentation, keypoint detection, and measurement. It is effective for measuring FMF angles in clinical work. The method proposed in this study reduces the subjectivity of sonographers in the diagnostic process and improves the efficiency and accuracy of the diagnosis.

## Figures and Tables

**Table 1:** Using DeepLabV3 to segment anatomical structure: IOU, PA, Dice.

Class	PA (%)	IOU (%)	Dice (%)
Palate	91.6	62.9	77.3
Frontal bone	95.1	70.3	82.5

**Table 2:** Five-fold cross validation with Dice.

Class	Fold 1 (%)	Fold 2 (%)	Fold 3 (%)	Fold 4 (%)	Fold 5 (%)	Average (SD) (%)
Palate	78.0	77.4	76.5	78.3	76.2	77.3 ± 1.830
Frontal bone	82.6	81.8	82.0	84.1	81.5	82.4 ± 0.923

**Table 3:** Comparison of our proposed method with the senior expert in the clinical validation set (mean ± std).

Group (*n* = 116)	FMF Angle (°)
AI group	67.352 ± 3.877
Senior expert	67.790 ± 4.609
Mean of difference	0.686 ± 2.85
MAE	2.354
MRE	0.035
*p*	<0.001

**Table 4:** Comparison of the junior expert and the senior expert in the clinical validation set (mean ± std).

Group (*n* = 116)	FMF Angle (°)
Junior expert	65.922 ± 4.363
Senior expert	67.790 ± 4.609
Mean of difference	−1.869 ± 3.967
MAE	3.428
MRE	0.050
*p*	<0.001

**Table 5:** Comparison of the reliability of FMF angle measurements between the junior expert and the senior expert.

Group (*n* = 116)	FMF Angle (°)	
	ICC	95% CI
AI and senior expert	0.760	0.666–0.829
Junior expert and senior expert	0.562	0.361–0.701

## Data Availability

The data presented in this study are available on request from the corresponding author.
